# *U-Bang-Haequi Tang*: A Herbal Prescription that Prevents Acute Inflammation through Inhibition of NF-**κ**B-Mediated Inducible Nitric Oxide Synthase

**DOI:** 10.1155/2014/542825

**Published:** 2014-05-15

**Authors:** Min Hwangbo, Ji Yun Jung, Sung Hwan Ki, Sang Mi Park, Kyung Hwan Jegal, Il Je Cho, Ju-Hee Lee, Seung Ho Kang, Sun-Dong Park, Sae Kwang Ku, Sang Chan Kim, Rong Jie Zhao, Seon Young Jee, Young Woo Kim

**Affiliations:** ^1^Department of Ophthalmology, Otolaryngology & Dermatology, Daegu Haany University, Daegu 706-828, Republic of Korea; ^2^Medical Research Center for Globalization of Herbal Formulation, College of Oriental Medicine, Daegu Haany University, Daegu 706-828, Republic of Korea; ^3^College of Pharmacy, Chosun University, Gwangju 501-759, Republic of Korea; ^4^Sunlin University, Pohang 791-712, Republic of Korea; ^5^College of Oriental Medicine, Dongguk University, Gyeongju 780-714, Republic of Korea; ^6^Department of Pharmacology, Mudanjiang Medical University, Mudanjiang 157011, China

## Abstract

Since antiquity, medical herbs have been prescribed for both treatment and preventative purposes. Herbal formulas are used to reduce toxicity as well as increase efficacy in traditional Korean medicine. *U-bang-haequi tang* (UBT) is a herbal prescription containing *Arctii fructus* and *Forsythia suspensa* as its main components and has treated many human diseases in traditional Korean medicine. This research investigated the effects of UBT against an acute phase of inflammation. For this, we measured induction of nitric oxide (NO) and related proteins in macrophage cell line stimulated by lipopolysaccharide (LPS). Further, paw swelling was measured in carrageenan-treated rats. Carrageenan significantly induced activation of inflammatory cells and increases in paw volume, whereas oral administration of 0.3 or 1 g/kg/day of UBT inhibited the acute inflammatory response. In RAW264.7 cells, UBT inhibited mRNA and protein expression levels of iNOS. UBT treatment also blocked elevation of NO production, nuclear translocation of NF-*κ*B, phosphorylation of I*κ*-B*α* induced by LPS. Moreover, UBT treatment significantly blocked the phosphorylation of p38 and c-Jun NH2-terminal kinases by LPS. In conclusion, UBT prevented both acute inflammation in rats as well as LPS-induced NO and iNOS gene expression through inhibition of NF-*κ*B in RAW264.7 cells.

## 1. Introduction

Inflammation is a crucial and physiological response of the immune system to infection [1–3]. This complex biological symptom is often caused by invading pathogens during harmful injury of human tissues [1–3]. In the inflammatory processes, an acute phase of inflammation is the initial responses, which include the increased movement of plasma and inflammatory cells from the blood stream to the tissue [1–3]. It has been shown that inflammation is related to the pathogenesis of various disorders such as metabolic disease, pulmonary disease, neoplasm, and infection [1–5]. Inflammatory reactions are mediated by signaling pathway in macrophage and mast cells and regulated by many factors (e.g., proinflammatory enzymes, cytokines, and other mediators) [6–9].

Lipopolysaccharide (LPS) is gram-negative bacteria and can activate macrophages as indicated by production of nitric oxide (NO), tumor necrosis factor-*α* (TNF-*α*), interleukins (ILs), and leukotrienes [10]. In fact, NO is a key mediator in the immunological defense and vasodilation. However, a large amount of NO can induce oxidative damage and nitrogen stress [11]. NO induced from L-arginine by NO synthase (NOS) enzymes in activated immune cells such as macrophages. NOSs are classified to three forms, that is, endothelial NOS (eNOS), neuronal NOS (nNOS), and inducible NOS (iNOS). Among them, iNOS produces high concentration of NO in specific tissue and mediates some autoimmune diseases such as chronic inflammation and sepsis [12].

TNF-*α* is known to play an important role as a macrophage activator and an initiator of immune response [13]. IL-6 is another crucial inflammatory cytokine and produced by macrophage cells [13]. It is also known to have a role in the acute phase of inflammation [13]. Nuclear factor-kappa B (NF-*κ*B) has been studied that it has proinflammatory activity by controlling inflammatory gene expressions [14]. In inactivated state, NF-*κ*B exists in cells as homodimeric or heterodimeric complexes and remains in the cytoplasm of cells by association with the NF-*κ*B inhibitory protein and inhibitory kappa B (I-*κ*B) [14]. However, once released from I-*κ*B*α* in the cytosol, NF-*κ*B can translocate into nuclei to activate the expression of proinflammatory genes such as iNOS, TNF-*α*, and IL-6 [15]. Prostaglandin E_2_ (PGE_2_) is also significant mediator which is produced from arachidonic acid metabolites [16].

*U-bang-haequi tang* (UBT) (*Niu Bang Jie Ji Tang* in Chinese) is a herbal prescription consisting of* Arctii fructus* and* Forsythia suspensa* as main components. In traditional Korean medicine, this herbal formula has been used for long period to treat a variety of diseases such as fever, infection, and inflammatory disorders. It is comprised mostly of cool plants that can remove pathogens from the body surface and alleviate swelling. To verify inhibitory effects of UBT on acute inflammation, we used the carrageenan-induced paw swelling animal model and activated macrophage cell model by LPS. In this study, we confirmed the effects of UBT against acute phase of inflammation in association with the inhibition of NF-*κ*B-mediated NO and PGE_2_ production.

## 2. Materials and Methods

### 2.1. Preparation of the UBT

The herbal components of UBT were purchased from Daewon pharmacy (Daegu, Korea) (Table 1). UBT was prepared by boiling 210 g of UBT in 1 L of water for 3 h and filtered by a 0.2 *μ*m filter (Nalgene, New York, NY, USA). It was lyophilized and stored at −20°C until use. The amount of UBT was estimated by the dried weight, and the yield was 18.75%.

### 2.2. Materials

Anti-I-*κ*B*α*, anti-p-I-*κ*B*α*, anti-iNOS, horseradish peroxidase-conjugated anti-mouse, anti-goat IgGs, and antibody for phosphorylation of mitogen-activated protein kinases (MAPKs) such as extracellular signal-regulated kinase (ERK), c-Jun NH_2_-terminal kinase (JNK), and p38 were purchased from Cell Signaling (Beverely, MA, USA). Anti-anti-NF-*κ*B p65, HSP 70 and Lamin A/C antibodies were supplied from Santa Cruz Biotechnology (Santa Cruz, CA, USA). Anti-murine iNOS was purchased from BD Bioscience (San Jose, CA, USA). PGE_2_ assay kit was supplied from R&D system (Minneapolis, MN, USA). TNF-*α* and IL-6 were supplied from Thermo Scientific (Waltham, MA, USA). Polyethylene glycol number 400 (PEG) solution, carrageenan, dexamethasone, and other reagents were purchased from Sigma Chemical Co. (St. Louis, MO, USA).

### 2.3. Carrageenan-Induced Paw Edema

Animal experiments were conducted under the guidelines of the Institutional Animal Care and Use Committee at Daegu Haany University (DHU). Sprague-Dawley rats at 4 weeks of age (male, 80–100 g) were provided from Hyochang Sience (Daegu, Korea) [17]. Rats (*N* = 25) were divided into five groups, and thus each group is five animals. UBT, dissolved in 40% of PEG number 400, was given to rats at the dose of 0.3 or 1 g/kg/day (p.o.) for 4 days. Dexamethasone (1 mg/kg/day) is a known anti-inflammatory drug and we used it as a positive control [18]. The paw volumes were measured up to 3 h after the carrageenan injection at intervals of 1 h. The hind paw volume was verified volumetrically by measuring using a plethysmometer (UGO BASILE, VA, Italy). After euthanasia, the paw samples were prepared.

### 2.4. Histological Process

The hind paw* dorsum* and* ventrum pedis* skins were fixed in 10% neutral buffered formalin, embedded in paraffin, sectioned (3~4 *μ*m), and stained with hematoxylin and eosin (H&E) [19]. The histopathological analysis of tissue samples was assessed under light microscope (Nikon, Japan).

### 2.5. Toluidine Blue Staining

The paw histological paraffin sections were deparaffinized. And then, they were stained with toluidine blue working solution for 2-3 minutes (1% toluidine blue contains 1% sodium chloride pH 2.2~2.5) and then dehydrated and mounted.

### 2.6. Histomorphometry

The thicknesses of* dorsum* and* ventrum pedis* skins (from epidermis to dermis; keratin layers were excluded) were measured using automated image analyzer (DMI-300 Image Processing; DMI, Korea) under magnification 40 of microscopy (Nikon, Japan) at prepare skin histological samples as mm/paw. The infiltrated inflammatory cells were also counted using automated image analyzer as cells/mm^2^ of dermis under magnification 200 of microscopy [19].

### 2.7. Cell Culture

Raw264.7 cell, a murine macrophage cell line, and THP-1 cells, a human monocytic cell line, were obtained from American Type Culture Collection (Rockville, MD, USA). Raw264.7 cells were incubated with 1 *μ*g/mL LPS (*Escherichia coli* 026:B6; Sigma, St. Louis, MO, USA). The cells were incubated without 10% fetal bovine serum for 24 h and then stimulated to LPS or LPS + UBT for the indicated time periods. UBT dissolved in distilled water was treated in medium 1 h before the addition of LPS.

### 2.8. MTT Cell Viability Assay

The cells were plated at a density of 1 × 10^5^ cells per well in a 24-well plate to determine any potential cytotoxicity [17]. Cells were treated with UBT for the 18 h. After incubation of the cells, live cells were stained with MTT (0.5 *μ*g/mL, 4 h). Produced formazan crystals were dissolved by 300 *μ*L dimethyl sulfoxide. Absorbance was measured at 540 nm using a Titertek Multiskan Automatic microplate reader (model MCC/340, Huntsville, AL, USA).

### 2.9. Assay of Nitrite Production

Raw264.7 cells were treated with UBT at the indicated doses for 1 h and continuously incubated with LPS (1 *μ*g/mL) for the next 18 h. NO production was monitored by measuring the nitrite content [17]. This was performed by mixing the culture medium with Griess reagent (1% sulfanilamide, 0.1% N-1-naphthylenediamine dihydrochloride and 2.5% phosphoric acid). The sample and reagent mixture were incubated in room temperature for 10 minutes. And then, the absorbance was assessed at 540 nm after 10 min.

### 2.10. Enzyme-Linked Immunosorbent Assay (ELISA)

To measure the TNF-*α*, IL-6 and PGE_2_ contents, Raw264.7 cells were incubated with UBT for 1 h and continuously stimulated with LPS for 12 h according to the manufacturer's instruction [16].

### 2.11. Immunoblot Analysis

Cells were lysed in the RIPA buffer [17]. Cell lysates were centrifuged at 15,000 g for 10 min to remove debris. The immunoreactive bands were visualized using enhanced chemiluminescence (ECL) detection kit (Amersham Biosciences Corp., Piscataway, NJ, USA) according to the manufacturer's instructions. Equal loading of proteins was verified by actin or Lamin immunoblottings. Nuclear extracts were prepared as previously described [18]. Intensity of the bands was determined using an image analyzing system (Ultra-Vilolet Products Lts., Upland, CA, USA). Repeated experiments were separately performed to confirm changes.

### 2.12. Real-Time RT-PCR Analysis

Total RNA was isolated from RAW264.7 using Trizol reagent (Invitrogen, Carlsbad, CA, USA). Real-time PCR was carried out according to the manufacturer's instructions [18]. We used a light cycler DNA master SYBR green-I kit (Light-Cycler 2.0, Roche, Mannheim, Germany). The relative levels of iNOS (sense: 5′-CCTCCTCCACCCTACCAAGT-3′, antisense: 5′-CACCCAAAGTGCTTCAGTCA-3′, 199 bp) were normalized based on the level of glyceraldehyde-3-phosphate dehydrogenase (sense: 5′-AACGACCCCTTCATTGAC-3′, antisense: 5′-TCCACGACATACTCAGCAC-3′, 173 bp).

### 2.13. Statistical Analysis

One-way analysis of variance (ANOVA) was used to analyze statistical significance of differences among each group. For assessing statistically significant effect, the Newman-Keuls test was used for comparisons between multiple group means. The data were expressed as means ±95% confidence intervals.

## 3. Results

### 3.1. Effect of UBT on Volume Changes in Carrageenan-Induced Paw Edema

**Figure 1:**
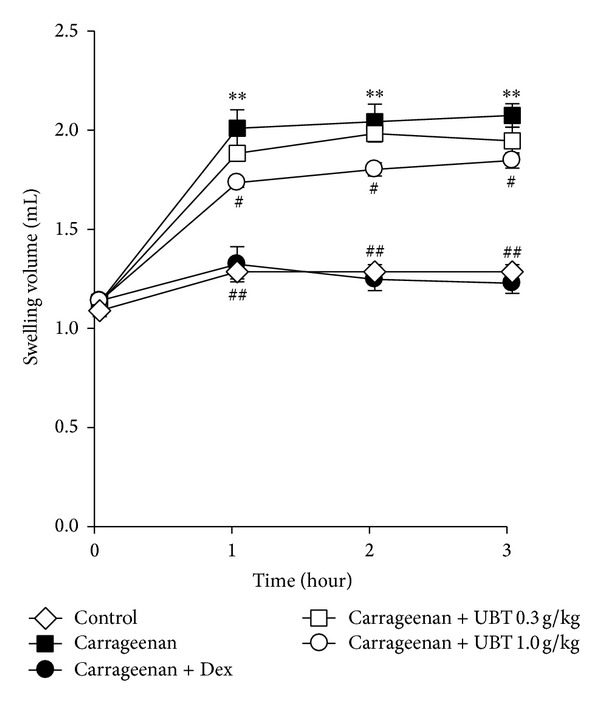
Inhibition of carrageenan-induced paw swelling by UBT. The UBT was administered to rats at the oral dose of 0.3 or 1.0 g/kg/day. The swelling volume of the paw was measured at 1–3 h after carrageenan injection. Dexamethasone (Dex, 1 mg/kg, p.o.) was used as a positive control. Data represents the mean ± S.E.M. of five animals (significant as compared with control, **P < 0.01; significant as compared with carrageenan alone, ^#^ *P* < 0.05, ^##^ *P* < 0.01). For data points where error bars could not be seen, the standard error was subtended by the data point. UBT,* U-bang-haequi tang*.

First, we showed the anti-inflammatory effect of UBT in the paw edema animal model. This carrageenan-induced paw swelling model is well known for screening the efficacy of drug candidates [20]. In this study, carrageenan injection successfully induced an increase of paw volume from 1 h. And this persisted up to 3 h after the injection (Figure 1). Oral administration of UBT 0.3 and 1 g/kg/day for 4 days inhibited the effect of carrageenan to induce paw swelling. We also compared positive control drug, dexamethasone (1 mg/kg/day, p.o., for 4 days), which also decreased the edema formation.

### 3.2. Effect of UBT in Carrageenan-Induced Acute Inflammation

**Figure 2:**
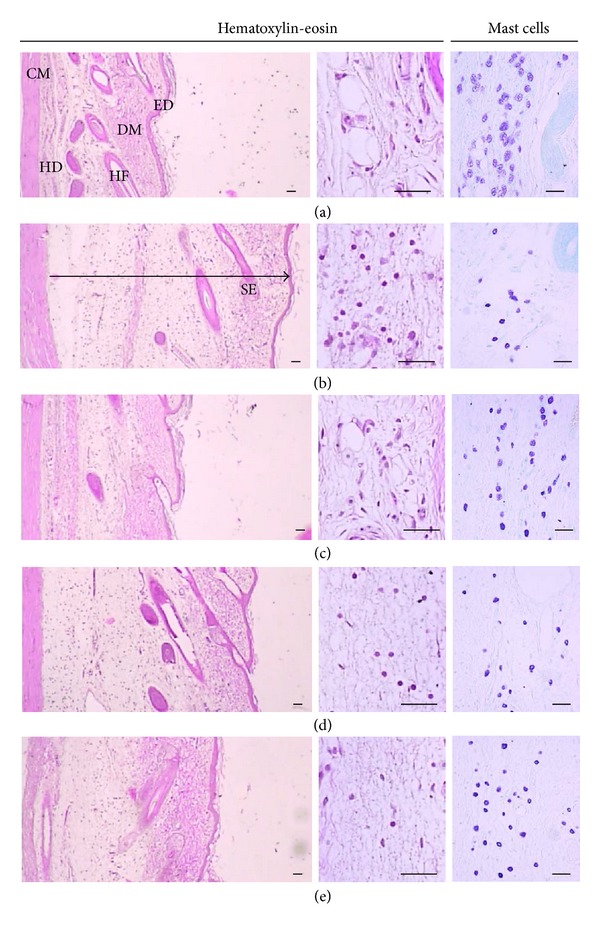
Histological profile of the* dorsum pedis* skin, taken from vehicle control (a), carrageenan control (b), dexamethasone (c), carrageenan + UBT 0.3 (d), and UBT 1.0 g/kg (e) treated rats. Histomorphometry of the* dorsum pedis* skin stained with H&E. Arrow indicates total thicknesses measured. Scale bars = 80 *μ*m. CA, carrageenan; ED, epidermis; DM, dermis; HD, hypodermis; PM, paw muscle.

**Figure 3:**
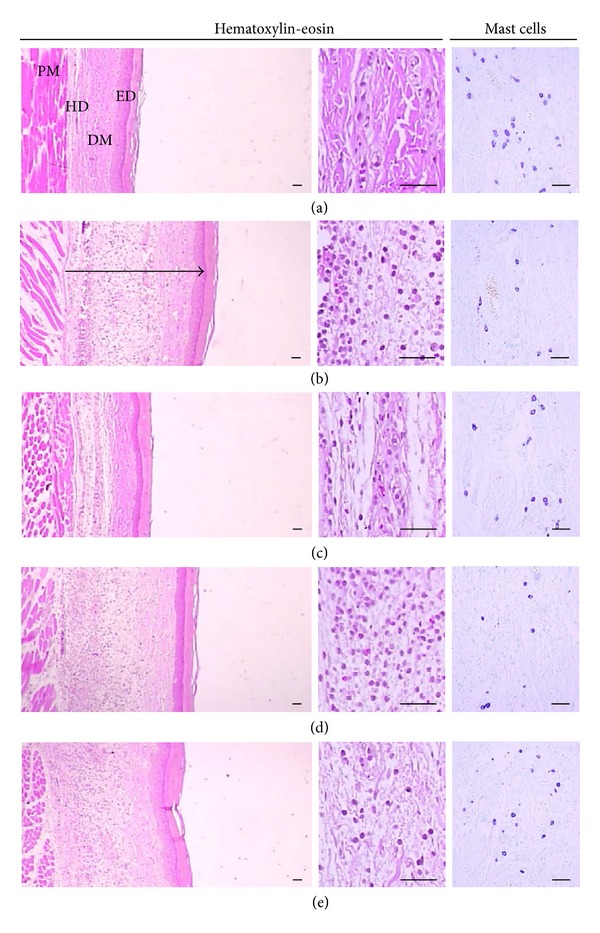
Histological profile of the* ventrum pedis* skin, taken from vehicle control (a), carrageenan control (b), dexamethasone (c), carrageenan + UBT 0.3 (d), and UBT 1.0 g/kg (e) treated rats. Histomorphometry of the* ventrum pedis* skin stained with H&E and used for histological samples. Arrow means total thicknesses. Scale bars = 80 *μ*m. CA, carrageenan; ED, epidermis; DM, dermis; HD, hypodermis; PM, paw muscle.

We assessed histological analysis of rat skin in* ventrum* and* dorsum pedis* by staining with H&E. As expected, carrageenan induced paw swelling in* ventrum* and* dorsum pedis* (Figures 2 and 3). Increase of the* ventrum* and* dorsum pedis* thicknesses was detected as results of acute edematous inflammation in carrageenan treated rats as compared with vehicle paw skins (Table 2). Carrageenan also induced increase of infiltrated inflammatory and degranulation-related decreases of mast cell, which are widely distributed through body and play a key role in inflammation (Table 2). Treatment of UBT (1.0 g/kg/day) significantly inhibited the mast cell degranulation and restored the number of mast cells as well as decreased the infiltration of inflammatory cells. All of these results suggest that UBT may have an anti-inflammatory effect in rats.

### 3.3. Inhibition of LPS-Inducible NO, PGE_2_ and Pro-Inflammatory Cytokines Production

**Figure 4:**
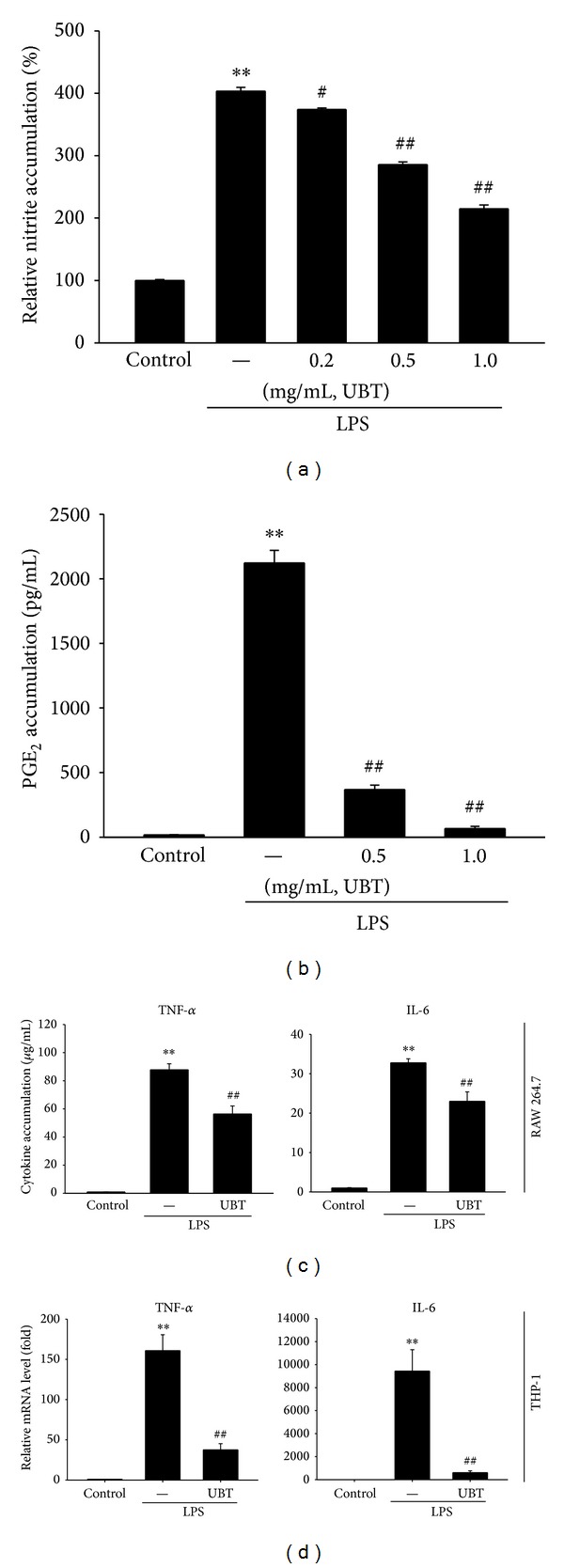
Inhibition of LPS-inducible NO and PGE_2_ by UBT. (a) NO and (b) PGE_2_ accumulations. Raw264.7 cells were treated with 0.2, 0.5, and 1.0 mg/mL of UBT for 1 h, and continuously with LPS (1 *μ*g/mL) for the next 18 h. (c) TNF-*α* and IL-6 contents in culture medium. Raw264.7 cells were stimulated by LPS with or without 1.0 mg/mL UBT for 12 h. (d) TNF-*α* and IL-6 mRNA levels. THP-1 cells were stimulated by LPS with or without 1.0 mg/mL UBT for 12 h. Data represents the mean ± S.E.M. from four separate experiments (significant as compared with control, **P < 0.01; significant as compared with LPS alone, ^#^ *P* < 0.05, ^##^ *P* < 0.01). UBT,* U-bang-haequi tang*.

In the next step, this study extended to effects of UBT on inflammation* in vitro*. We evaluated cellular toxicity of UBT in Raw264.7 cells. In MTT assay, cell survival was not affected by UBT treatment 0.5 and 1.0 mg/mL (i.e., control, 100 ± 3.6%; UBT 0.5 mg/mL, 101 ± 2.3%; UBT 1.0 mg/mL 107 ± 4.6%). Next, we determined the effect of UBT on NO production in LPS-induced Raw264.7 cells. As expected, LPS (1 *μ*g/mL, 18 h) increased NO accumulation, which was significantly reduced by UBT (0.2, 0.5 and 1.0 mg/mL) in a concentration-dependent manner (Figure 4(a)). LPS-stimulated PGE_2_ production in Raw264.7 cells was also blocked by UBT (0.5 and 1.0 mg/mL) (Figure 4(b)).

To confirm the effect of UBT on cytokines, we measured TNF-*α* and IL-6 production in Raw264.7 cells by using ELISA assay. LPS-inducible increased the productions of TNF-*α* and IL-6 in culture media of Raw264.7 cells (Figure 4(c)). Treatment of UBT (1.0 mg/mL) decreased the ability of LPS (1 *μ*g/mL, 12 h) to increase the cytokines accumulation (Figure 4(c)). Moreover, we additionally determined the effects of UBT on TNF-*α* and IL-6 in THP-1 cells. UBT pretreatments completely inhibited increase in the relative mRNA expression of TNF-*α* and IL-6 elicited by LPS (Figure 4(d)).

### 3.4. Effect of UBT on the LPS-Induced iNOS Gene Expression

**Figure 5:**
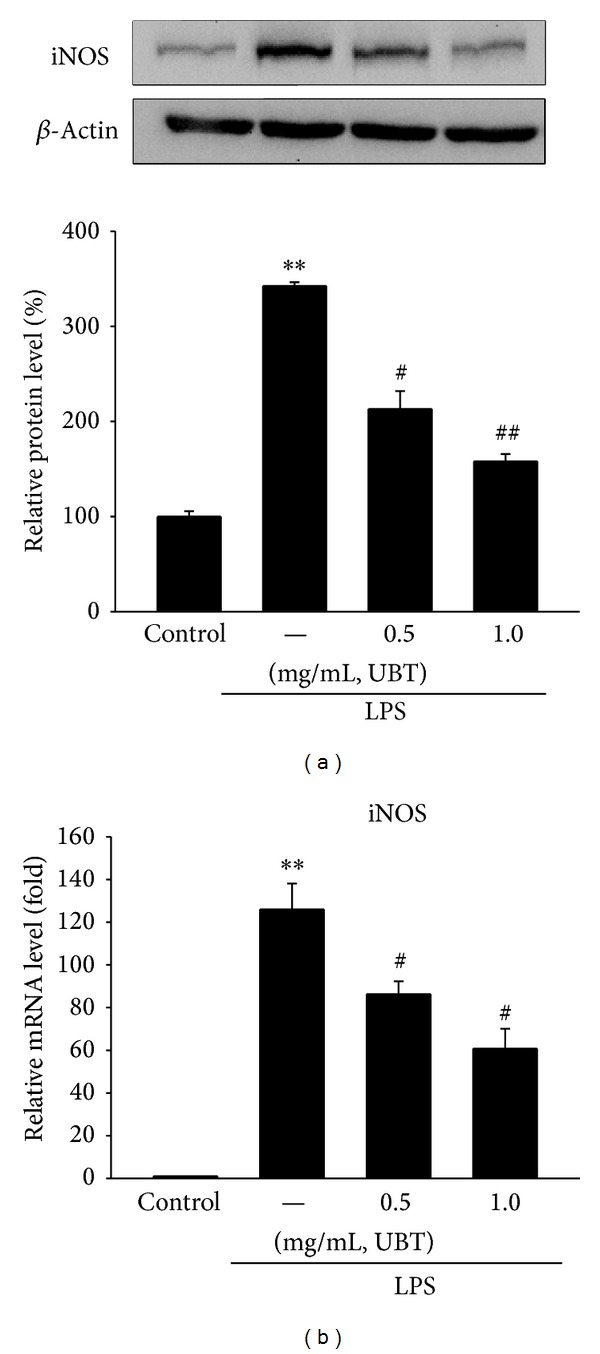
Inhibition of LPS-inducible iNOS gene expression by UBT. (a) Representative iNOS immunoblottings. (b) Relative iNOS mRNA levels. The expressions of protein and mRNA were assessed 18 h after treatment with LPS + UBT. Data represents the mean ± S.E.M. from three experiments (significant as compared with control, **P < 0.01; significant as compared with LPS alone, ^#^ *P* < 0.05, ^##^ *P* < 0.01). UBT,* U-bang-haequi tang*.

Next, we assessed immunoblot analysis to verify the protein expressions of iNOS. Raw264.7 cells were pretreated with UBT (0.5 and 1.0 mg/mL) for 1 h and LPS treatment at 1 *μ*g/mL for 18 h dramatically increased protein levels of iNOS (Figure 5(a)). Densitometer analysis revealed that UBT treatment significantly inhibited the iNOS protein expressions in Raw264.7 cells (Figure 5(a)). Moreover, real-time RT-PCR analysis verified the effect of UBT on iNOS mRNA expression. UBT significantly repressed the increase of its mRNA by LPS, which implies that UBT can downregulate the gene induction of iNOS (Figure 5(b)).

### 3.5. Effect of UBT on LPS-Inducible Degradation of I-*κ*B*α* and Phosphorylation of MAPKs

**Figure 6:**
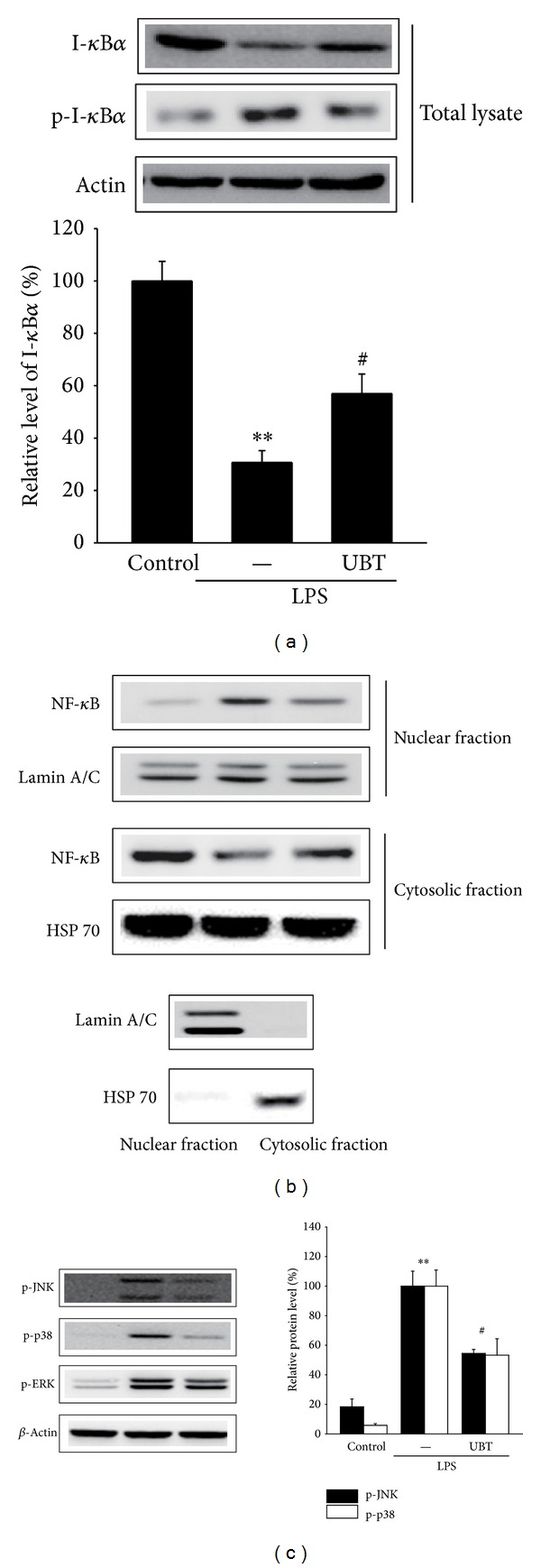
Effect of UBT on LPS-inducible degradation of I-*κ*B*α* and phosphorylation of mitogen-activated protein kinases (MAPKs). (a) The level of I-*κ*B*α* and p-I-*κ*B*α* in total lysates. Raw264.7 cells were stimulated with LPS or LPS + UBT for 30 min. Immunoblots are representative of results from repeated experiments. (b) Nuclear and cytosolic level of NF-*κ*B. Lamin A/C and HSP 70 confirmed equal loading and purity of the each fraction. (c) The phosphorylation c-Jun NH_2_-terminal kinase (JNK), p38, and extracellular signal-regulated kinase (ERK) in total lysates. Raw264.7 cells were incubated with LPS ± UBT for 30 min. For A and C, data represents the mean ± S.E.M. from three separate experiments (significant as compared with control, **P < 0.01; significant as compared with LPS alone, ^#^ *P* < 0.05). JNK, c-Jun NH_2_-terminal kinase; ERK, extracellular signal-regulated kinase; MAPK, mitogen-activated protein kinase; UBT,* U-bang-haequi tang*.

NF-*κ*B serves as the important transcription factor for the induction of the proinflammatory genes in microphage cells stimulated by inflammatory inducers such as LPS [20–22]. NF-*κ*B translocates to the nucleus by phosphorylation of I-*κ*B*α* (p-I-*κ*B*α*) and subsequent degradation of I-*κ*B*α* subunit. Exposure of LPS decreased the protein level of p-I-*κ*B*α* and I-*κ*B*α* at 30 min. Treatment of Raw264.7 cells with UBT for 1 h inhibited the phosphorylation and degradation of I-*κ*B*α* induced by LPS (Figure 6(a)). Moreover, UBT also blocked the translocation of nuclear NF-*κ*B by LPS treatment (Figure 6(b)). These results suggest that UBT might prevent translocation of NF-*κ*B to the nucleus by phosphorylation and degradation of I-*κ*B*α*.

MAPKs, the serine/threonine protein kinases, consisted of three family members (i.e., JNK, p38, and ERK) and play key roles in regulation of the inflammatory responses by leading to activation of NF-*κ*B and induction of proinflammatory genes [23]. To investigate the upstream molecular target of UBT, we assessed the effects of UBT on LPS-induced phosphorylation of JNK, p38, and ERK. As expected, treatment of LPS upregulated phosphorylation of JNK, p38, and ERK. However, cotreatment of UBT resulted in significantly decreased phosphorylation of JNK and p38, but not ERK (Figure 6(c)).

## 4. Discussion

A medical herb has been mixed with other herbs, which is called a herbal prescription, and this mixture of various medical herbs is necessary for reducing toxicity and potentiating efficacy in traditional medicine [24]. UBT prescription is composed of 9 medical herbs including of* Fructus Arctii*,* Herba Menthae*,* Fructus Gardeniae*,* Herba Dendrobii*,* Herba Schizonepetae*,* Fructus Forsythiae*,* Radix Scrophulariae*,* Cortex Moutan Radicis*, and* Spica Prunellae *(Table 1). In traditional Eastern medicine, this prescription comprises cool ingredients that can treat pathogens from the body surface and alleviate phlegm and has been used to treat a variety of diseases such as early-stage infections in head and neck regions with obvious redness, swelling, heat, and pain [24]. Recent paper has reported that UBT has antiallergic effects in the contact dermatitis model [25]. Nevertheless, the scientific proof and mechanistic basis for the effect of UBT have almost not been elucidated. Here, we determined anti-inflammatory effects of UBT* in vivo* and* in vitro*.

Swelling is a major characteristic of acute inflammation cause by increase vascular permeability [26]. Therefore, induction of edema by carrageenan is a well-established model screening the effects of novel anti-inflammatory drugs [19, 27, 28]. Here, carrageenan successfully induced acute inflammation in the aspect of paw swelling and immunohistochemical analysis in the tissue stained with H&E [29]. Oral administration of UBT significantly decreased carrageenan-increased paw volume and inflammatory cell infiltrations. Moreover, it has been shown that NO and PGE_2_ might have important function in development of hyperalgesia in the process of inflammatory response and tissue injury [30–32]. These results demonstrated that UBT attenuates the acute the inflammation in rats.

NO is a significant regulator in the processes of pathophysiological symptoms (e.g., inflammation). A small amount of NO has beneficial functions including regulation of immune mechanism, vasodilation, and platelet inhibition. However, a high level of NO could induce damage to normal cell. LPS is known to activate macrophages to induce proinflammatory mediators, and NO is a hall marker of activation of immune cell in acute inflammation [33, 34]. In this study, we confirmed the effect of UBT on production of NO in LPS-inducible RAW264.7 cells. Treatment of UBT significantly inhibited production of NO and PGE_2_, another marker of inflammation. Moreover, UBT markedly blocked the iNOS protein levels, showing that UBT may have the effects on NO production by LPS through suppression of iNOS gene.

Cytokines play a significant role in inflammation disease to activate macrophage [35]. TNF-*α* was determined as a main inflammation mediator and an initiator of immune response [35]. TNF-*α* is produced by immune cells and could increase the expression of other cytokines such as ILs [36]. IL-6 is also one of the most important mediators of initiating the synthesis of PGE_2_. In this study, we determined the level of cytokines in media of RAW264.7 cells and confirmed the effects of UBT on cytokine level, showing that UBT has an ability of inhibiting acute inflammation* in vivo *and* in vitro*.

NF-*κ*B is a transcription factor controlling of the gene expressions involving inflammation, immune responses, and survival [21, 22, 37]. It is well established that drug candidates for the inflammation have inhibitory effects on nitric oxide, eicosanoids, and cytokines by suppressing NF-*κ*B activation [38]. Moreover, it has been shown that the promoters of iNOS and TNF-*α* include the binding sites for transcription factors, among which NF-*κ*B is known as one of the most important factors for their gene transcription. In fact, activation of NF-*κ*B transcriptionally induces the expression of various inflammatory mediators [39, 40]. The degradation of I-*κ*B*α* by phosphorylation causes activation and resultantly translocation of NF-*κ*B to nucleus. Our results verify that UBT blocked accumulation of NO and related gene expression through inhibition of I-*κ*B*α*-NF-*κ*B pathway.

## 5. Conclusion

In this study, we confirmed anti-inflammatory effects of UBT* in vivo* and* in vitro*. We used two approaches to show the effect of UBT: (1) an animal model, a carrageenan-induced acute inflammation, and (2) a cell model, an activated macrophage induced by LPS. In rats, UBT reduced inflammatory responses in carrageenan-induced paw swelling model. In macrophages, UBT has inhibitory effects on NO, PGE_2_, and cytokines as well as gene expression of iNOS. More importantly, the suppression of acute inflammation by UBT might result from its inhibition of NF-*κ*B activation by blocking degradation of I-*κ*B*α* as mediated with inhibition of JNK and p38. The findings here might help to understand the pharmacological effects and mechanism of the UBT and suggest the possibility for the treatment of inflammatory disease as a herbal formula.

## Figures and Tables

**Table 1:** Herbal components in *U-bang-haequi tang *(UBT).

Name of medical herbs	Contents in the prescription (g)
*Fructus Arctii *	10
*Fructus Forsythiae *	12
*Herba Dendrobii *	12
*Herba Menthae *	10
*Fructus Gardeniae *	10
*Radix Scrophulariae *	10
*Herba Schizonepetae *	6
*Cortex Moutan Radicis *	10
*Spica Prunellae *	12

Total	92

**Table 2:** Changes on the histomorphometrical analysis of hind paw skins [group summary].

Groups	Skins index					
	*Dorsum pedis* skin			*Ventrum pedis* skin		
	Total thickness (*μ*m)	IF cell numbers (cells/mm^2^)	Mast cell numbers (cells/mm^2^)	Total thickness (*μ*m)	IF cell numbers (cells/mm^2^)	Mast cell numbers (cells/mm^2^)
Control						
Intact	1219.09 ± 99.09	35.60 ± 16.68	60.40 ± 9.50	624.73 ± 64.76	15.60 ± 3.36	43.20 ± 10.35
CA	2401.20 ± 327.10^d^	276.00 ± 40.68^a^	21.80 ± 3.83^a^	1630.35 ± 158.60^a^	558.60 ± 56.81^a^	10.80 ± 2.39^a^
Reference						
Dexamethasone	1309.67 ± 171.86^de^	94.60 ± 14.21^ab^	45.60 ± 6.02^ab^	709.26 ± 105.95^b^	173.80 ± 47.01^ab^	24.60 ± 3.78^ab^
Test material (g/kg)						
UBT 0.3	1931.59 ± 217.43^df^	227.60 ± 24.72^ab^	28.40 ± 6.62^a^	1345.74 ± 76.23^ab^	568.60 ± 55.61^a^	11.40 ± 2.30^a^
UBT 1.0	1739.32 ± 176.30^df^	169.80 ± 12.54^ab^	36.00 ± 5.10^ab^	1285.90 ± 100.10^ab^	378.00 ± 79.16^ab^	18.00 ± 1.58^ac^

Values are expressed as mean ± SD of five rat hind paws.

CA: carrageenan.

IF: infiltrated inflammatory cells.

^a^ *P* < 0.01 as compared with intact control by LSD test.

^b^ *P* < 0.01 and ^c^ *P* < 0.05 as compared with CA control by LSD test.

^d^ *P* < 0.01 as compared with intact control by MW test.

^e^ *P* < 0.01 and ^f^ *P* < 0.05 as compared with CA control by MW test.

## Abbreviations

ERK: Extracellular signal-regulated kinase
ILs: Interleukins
iNOS: Inducible nitric oxide synthase
I-*κ*B: Inhibitory kappa B
JNK: c-Jun NH_2_-terminal kinase
LPS: Lipopolysaccharide
MAPKs: Mitogen-activated protein kinases
NF-*κ*B: Nuclear factor-kappa B
PGE_2_: Prostaglandin E_2_
TNF-*α*: Tumor necrosis factor-*α*.
UBT: * U-bang-haequi tang*
